# Towards the Burden of Human Leptospirosis: Duration of Acute Illness and Occurrence of Post-Leptospirosis Symptoms of Patients in The Netherlands

**DOI:** 10.1371/journal.pone.0076549

**Published:** 2013-10-03

**Authors:** Marga G. A. Goris, Vanessa Kikken, Masja Straetemans, Sandra Alba, Marco Goeijenbier, Eric C. M. van Gorp, Kimberly R. Boer, Jiri F. P. Wagenaar, Rudy A. Hartskeerl

**Affiliations:** 1 WHO/FAO/OIE and National Leptospirosis Reference Center and section Epidemiology, Royal Tropical Institute, KIT Biomedical Research, Amsterdam, The Netherlands; 2 Athena Institute, Free University, Amsterdam, The Netherlands; 3 Department of Virology, Erasmus Medical Center, Rotterdam, The Netherlands; University of Kentucky College of Medicine, United States of America

## Abstract

**Background:**

Leptospirosis is a global zoonotic disease. Although important for the assessment of the burden of leptospirosis, data on the duration of the illness and the occurrence of post-leptospirosis complaints are not well documented. Hence the main objective of this study was to estimate the occurrence of persistent complaints and duration of hospital stay in laboratory confirmed leptospirosis patients in the Netherlands during 1985 to 2010. Additionally, several risk factors potentially impacting on the occurrence of post-leptospirosis complaints were investigated.

**Methods/Principal Findings:**

The duration of the acute phase of leptospirosis was 16 days (IQR 12–23); 10 days (IQR 7–16) were spent hospitalized. Eighteen fatal cases were excluded from this analysis. Complaints of leptospirosis patients by passive case investigations (CPC) derived from files on ambulant consultations occurring one month after hospital discharge, revealed persistent complaints in 108 of 236 (45.8%) laboratory confirmed cases. Data on persistent complaints after acute leptospirosis (PCAC), assessed in 225 laboratory confirmed leptospirosis cases collected through questionnaires during 1985-1993, indicated 68 (30.2%) PCAC cases. Frequently reported complaints included (extreme) fatigue, myalgia, malaise, headache, and a weak physical condition. These complaints prolonged in 21.1% of the cases beyond 24 months after onset of disease. There was no association between post-leptospirosis complaints and hospitalization. However, individuals admitted at the intensive care unit (ICU) were twice as likely to have continuing complaints after discharge adjusting for age and dialysis (OR 2.0 95% CI 0.8-4.8). No significant association could be found between prolongation of complaints and infecting serogroup, although subgroup analysis suggest that infection with serogroups Sejroe (OR 4.8, 95%CI 0.9-27.0) and icterohaemorrhagiae (OR 2.0, 95%CI 0.9-4.3 CI) are more likely to result in CPC than infections with serogroup Grippotyphosa.

**Conclusion/Significance:**

In addition to the acute disease, persistent complaints have an impact on the burden of leptospirosis.

## Introduction

Leptospirosis is a zoonotic disease with worldwide distribution [1]. Human leptospirosis is a neglected and underreported disease [2], due to a lack of awareness, misdiagnosis and technically demanding laboratory confirmation tests [2,3]. Recent international initiatives on the assessment of the global disease burden of leptospirosis have estimated the global annual incidence of endemic human leptospirosis exceeding 5 severe cases per 100.000 population, excluding cases from outbreaks [2]. However, this number probably presents a substantial underestimation; as this incidence is derived from underreported, laboratory confirmed cases. The true total incidence of mild and severe leptospirosis most likely presents only a small portion of the true number of mild to severe cases annually.

Leptospirosis is caused by pathogenic spirochaetes of the genus *Leptospira* [4]. A wide variety of mammalian hosts, both feral and (semi-) domestic, serve as infection reservoirs. In developing countries, urban leptospirosis is an increasing public health hazard due to hygiene and overcrowding issues, however, farmers still represent the major risk group for leptospirosis [5]. In developed, temperate countries leptospirosis is mainly a recreational disease, associated with water-contact activities [1,2].

Leptospirosis is a protean disease ranging from a sub-clinical illness to a potentially fatal disease with haemorrhage and multi-organ failure as its clinical hallmarks. Persistent or chronic symptoms are often variable in character and onset [6]. This complicates the association between acute disease and sequelae [6,7] and makes it difficult to truly measure the burden of disease. Apart from some anecdotal case reports, post-leptospirosis complaints are not well assessed [3]. Data on the impact and occurrence of persistent or chronic complaints, together with data on the period of hospitalization are badly needed for assessing the burden of leptospirosis in terms of disability-adjusted life years (DALYs) [2,3].

Therefore, in this paper we describe the estimate of the burden of hospitalized and outpatient human leptospirosis in The Netherlands with regard to the duration of the acute and chronic illness as well as the incidence of persistent symptoms by active and passive case finding. We estimate the effect of intensive care unit (ICU) stay adjusted for the potential confounding effect of age, gender, infecting serogroup and occurrence of the acute severe complications of lung (acute lung injury, (ALI)) and kidney (acute renal injury (ARI)), liver injury and haemorrhage. As well, several studies have shown that ICU admission is a risk factor for both physical and psychological symptoms up to 12 months after discharge from the hospital [8]. Psychological consequences include depressive symptoms, post-traumatic stress disorders (PTSD), anxiety and chronic fatigue syndrome (CFS). These sequelae were associated with traumatic memories during ICU or hospital stay [9–11]. In the light of these studies regarding persistent symptoms, in this study it was considered of particular importance, to determine whether there is an association between ICU stay and persistent symptoms after the acute phase of leptospirosis.

## Methods

### Ethical statement

The Medical Ethical Review Committee of the Academic Medical Center, University of Amsterdam provided clearance for the execution of the active survey on persistent complaints (96.17.038). In addition, the Medical Ethical Review Committee waived the study for human subjects research ethical review for the data collected from passive case finding (W12_075#12.17.0092). All presented data have been de-identified and are not attributable to individual patients.

### Study participants and data collection

The WHO/FAO/OIE and National Leptospirosis Reference Center (NRL) resides in the department of Biomedical Research of the Royal Tropical Institute in Amsterdam, The Netherlands. NRL forms the diagnostic center for leptospirosis in the Netherlands, confirming nearly 100% of the cases suspected of leptospirosis in the country. Confirmation was done in accordance with the national laboratory case definition [12], which is consistent with the definition of the WHO-Leptospirosis Epidemiology Reference Group (LERG) [2].

From 1985 until 2010 the laboratory received an estimated 20,000 samples for investigation. Detailed records on serological, clinical and epidemiological data, including letters of discharge and ambulant consultations after discharge from the hospitals are archived in the NRL. Abstracted data from this archive were used to assess post-leptospirosis complaints by active and passive case finding (see below), duration of illness, hospitalization and attendance of the ICU. Data on the health status of the general population in the Netherlands were retrieved from the Dutch Central Bureau for Statistics (CBS).

### Persistent Complaints by Active Case finding

In 1997 an active survey on long-term persistent symptoms among leptospirosis confirmed patients was conducted. Questionnaires were sent to the general practitioners (GP) of all confirmed cases from the years 1985 to 1993. Complaints derived from the questionnaires are referred to as Persistent Complaints by Active Case finding (PCAC) and comprises both hospitalized patients and outpatients. The following questions were included in the questionnaire: (i) Did the patient have long-term complaints after the acute disease? (ii) If so, what were the complaints? (iii) What was the duration of these complaints?

### Complaints determined by Passive Case finding

Complaints by passive case (CPC) finding were collected from all hospitalized leptospirosis patients from 1985 to 2010 that were confirmed by NRL. Archived data [13] included information on symptoms and signs, including those from ambulant consultations. These were usually collected one month after patient discharge from the hospital. For the purpose of determining continued reported complaints following hospital discharge this data was compiled, entered and analysed.

### Data processing and definitions

Fatality was used as an exclusion criterion in the analyses for several reasons; firstly, progression towards death is usually rapid and not a good indicator for the mean duration of a stay in the hospital and secondly, death itself is a major burden factor.

The total acute phase of illness is defined as the first day of illness till the day of discharge from the hospital, which includes the date of discharge. The total acute phase of illness was separated into number of days prior to hospital admission and number of days of hospital stay. ICU stay was defined as the first day of admittance to discharge from ICU. If either of these dates were missing, ICU stay was recorded as yes, but the length of time was not calculated and recorded as missing. Cases with incomplete data affecting a specific evaluation (for example, lacking data on the duration of hospital stay) were excluded only from the part of the analyses. Also, questionnaires that contained conflicting or incomplete information were excluded from the analyses.

Other factors that were included in the analyses were gender and age (<38 years; ≥38 years) in which 38 years of age was the median age in the study population during the acute illness. If information was recorded concerning difficulties at work it was included in the data analyses. Data on the infecting serogroup were deduced from the serovar giving the highest titers in the MAT [14]. Common complaints were classified as separate variables in the database. Data was collected on liver injury, haemorrhage, acute renal injury (ARI) and acute lung injury (ALI). WHO-LERG definitions were used for ARI and ALI [2]. In brief, ALI patients presents with respiratory distress indicated by dyspnoea and/or a respiratory frequency ≥28 per minute, bilateral crepitus, bilateral infiltrates in chest X-ray examination, a ratio of the partial pressure of arterial oxygen to the fraction of inspired oxygen (PaO2/FiO2) <300mm or report of the use of mechanical ventilation as a therapeutic intervention. ARI is defined by an acute onset of oliguria, uraemia, or abnormally elevated serum creatinin or blood urea nitrogen. Liver injury is defined by jaundice, hepatitis and/or abnormal laboratory findings with respect to blood albumin and/or bilirubin, alanine-aminotransferase (ALAT or SGPT), aspartate-aminotransferase (ASAT or SGOT) and/or lactate dehydrogenase (LDH), alkaline phosphatase and γ-glutamyl transpeptidase (γ-gt). The term ‘haemorrhage’ refers to a recorded bleeding such as petechiae and gastrointestinal bleeding.

### Data analysis

Descriptive data is presented on the duration of acute illness, the presence of complaints after discharge from the hospital in the CPC group and presence, nature and duration of complaints in the PCAC group using median (IQR) and proportions. Mann Whitney tests were conducted to determine differences in duration of acute illness by age and sex. Chi-square tests and fisher’s exact tests were used to assess the association between reported complaints by age, gender, infecting serogroup and occurrence of the acute severe complications ALI, ARI, liver injury and haemorrhage and ICU stay.

Multivariate logistic regression analysis was performed to assess risk factors for ICU stay. Variables were considered for the multivariate model if it was associated with complaints and ICU stay with a p<0.10. Associations are presented by Odds Ratios (OR) with the corresponding 95% confidence intervals. Analyses were conducted using SPSS software version 17.0.

## Results

### Incidence and duration of illness

During the period 1985 to 2010, 829 patients were confirmed with leptospirosis; 764 (92.3%) males and 64 (7.7%) females (Table 1). A total of 570 patients (78.9%) were known to be hospitalized, 106 patients (of whom 13 died) were reported to have been admitted to the ICU (Table 1). Reference to the ICU has been poorly documented, but from those with recorded data, ICU admission was usually within the first day of hospital admission.

**Table 1:** Patient characteristics of leptospirosis patients separated according to available data*.

Characteristic		Hospitalized and outpatients	Hospitalized patients	Hospitalized patients	Hospitalized and outpatients
		All laboratory confirmed patients	Days of acute illness known	Health status after discharge known; Complaints by Passive Case Finding Group	Eligible questionnaires; Persistent Complaints by Active Case finding Group
period		1985-2010	1985-2010	1985-2010	1985-1993
n		829	369	236	225
Male†		764 (92.3)	332 (90.5)	213 (90.6)	214 (95.5)
Age - median (25^th^ and 75^th^ percentile)		38 (26-52)	38 (25-51)	37 (25-50)	38 (27-51)
Antibiotic treatment **		653 (86.0)	327 (89.1)	205 (86.9)	171 (83.8)
Hospital admission#		570 (78.9)	368 (100)	236 (100)	129 (73.7)
Deceased no. (%)##		19 (2.3)	0	0	0
Dialysis on hospitalized patients††		79 (16.1)	46 (12.7)	24 (10.2)	20 (17.9)
ICU admission***		106 (21.3)	52 (14.5)	31 (13.4)	11 (10.3)
Serogroup known		676	298	192	205
	Missing	153	71	44	20
Icterohaemorrhagiae		312 (46.4)	154 (51.7)	92 (47.9)	84 (41.0)
Grippotyphosa		114 (16.9)	57 (19.1)	39 (20.3)	33 (16.1)
Sejroe		110 (16.3)	18 (6.0)	8 (4.2)	67 (32.7)
Pomona		56 (8.3)	28 (9.4)	18 (9.4)	14 (6.8)
Other serogroups		84 (12.4)	41 (13.8)	35 (18.2)	7 (3.4)
**Post-leptospirosis complaints**					
Occurrence CPC		NA	NA	108 (45.8%)	NA
Occurrence PCAC^00^		NA	NA	NA	68 (30.2%)
Extreme fatigue		NA	NA	NA	44 (67.7%)
Myalgia		NA	NA	NA	13 (20.0%)
Malaise		NA	NA	NA	11 (16.9%)
Headaches		NA	NA	NA	10 (15.4)
‘Hampered’ physical condition		NA	NA	NA	10 (15.4%)
Joint complaints		NA	NA	NA	6 (9.2%)
Disability to work		NA	NA	NA	9 (13.8%)
Others		NA	NA	NA	27 (41.5%)
**Acute injuries**					
Period		1985-1993	1985-1993	1985-1993	1985-1993
n		332	125	62	225
ARI	Yes	165 (61.8)	95 (78.5)	49 (83.1)	111 (59.7)
	No	102 (38.2)	26 (21.5)	10 (16.9)	75 (40.3)
	Missing	65	4	3	39
Haemorrhage	Yes	62 (31.0)	36 (38.7)	19 (38.0)	43 (29.9)
	No	138 (69.0)	57 (61.3)	31 (62.0)	101 (70.1)
	Missing	132	32	12	81
Liver injury	Yes	184 (80.7)	91 (81.3)	46 (80.7)	128 (79.5)
	No	44 (19.3)	21 (18.8)	11 (19.3)	33 (20.5)
	Missing	104	13	5	64
ALI	Yes	27 (19.7)	15 (14.2)	7 (13.7)	15 (16.1)
	No	110 (80.3)	91 (85.8)	44 (86.3)	78 (83.9)
	Missing	195	19	11	132

NA Is Not Applicable

Values are no. (%) unless otherwise indicated

† Sex was registered for 828 patients

** Data on antibiotic treatment available from respectively 759/829, 367/368, 236/236 and 204/225 patients

Data on hospital admission available from respectively 722/829, 368/368, 236/236 and 175/225 patients

## 18 Patients died in the hospital, 1 patient is unknown

†† Data on dialysis on hospitalized patients available from respectively 491/570, 361/368, 235/236 and 112/129 patients

*** Data on ICU admission on hospitalized patients available from respectively 497/570, 359/368, 231/236 and 107/122 patients

^00^ Symptoms for PCAC available for 65 cases

18 males and 1 female died during the recorded period (Table 1). Based on available data of 369 (67%) of the 552 surviving hospitalized patients the median duration of total acute phase of illness was 16 days (IQR 12–23 days). The median duration of illness prior to hospital admission was 5 days and of hospital stay was 10 days. The median duration of ICU stay was 7 days (Table 2).

**Table 2:** Days of acute illness of hospitalized, surviving patients in the period 1985 to 2010, stratified according to gender and age.

**Duration in Category**	**Number of patients**	**Median (25^th^ and 75^th^ percentile)**	P-value*
Acute disease total	369	16 (12-23)	NA
Males	333	16 (12-23)	0.018
Females	35	13 (9-21)	
Age <38 years	185	14 (11-19)	<0.001
Age ≥38 years	187	19 (14-25)	
Prior hospitalization total	390	5 (3-7)	NA
Males	352	5 (3-7)	0.735
Females	37	5 (2-7)	
Age <38 years	191	4 (3-7)	0.066
Age ≥38 years	199	5 (4-7)	
Hospitalization total	366	10 (7-16)	NA
Males	331	11 (7-16)	0.013
Females	34	7.5 (6-12.5)	
Age <38 years	182	9 (6-13)	<0.001
Age ≥38 years	184	12 (8-18)	
ICU attendance total	20	7 (4-13.5)	NA
Males	19	7 (4-14)	0.223
Females	1	3	
Age <38 years	7	8 (3-12)	0.937
Age ≥38 years	13	13 (4-14.5)	

NA is Not applicable

* Mann Whitney test

Current policies in the Netherlands aim at reducing the period of hospitalization. Consistently, in the years 2005 to 2010 the median duration of hospital attendance was lower (8 days IQR 6–11 days) compared to the entire period 1985-2010 (10 days; IQR 7–16 days) while the period ‘prior hospitalization’ remained virtually the same (median 4 days, IQR 3-7 days).

The median number of days prior to hospital admission did not differ by age (<38 and ≥38 years) or gender. However, males had a longer hospital stay compared to females (11 versus 7.5 day, p=0.013). Individuals aged ≥ 38 had longer hospital stay (12 versus 9 days, p<0.001).

### Factors associated with Complaints by Passive Case finding (CPC)

Data was available on health status after discharge for 236 of the 552 hospitalized surviving patients, with a small difference in age and gender of those with data available and not available. The number of males in the included versus the excluded cases was 213 (90.6%) and 295 (93.4%), respectively and the median age was 37 years, (IQR 25-50) for those included and 39 years, (IQR 26-54) for those excluded.

The number of CPC was 108 (45.8%) (Table 1). Individuals of older age (≥ 38 years) were two times more likely to have CPC compared to individuals of younger age (OR 2.2, 95%CI 1.3-3.7) (Table 3). Complaints typically comprised non-specific aberrations noticed during physical examination during the follow-up visit by physician, such as (extreme) fatigue, headaches, hair loss at young age, mild jaundice and disability to work and/or supported by abnormal laboratory findings. No association was found between CPC and gender (males, OR 1.3, 95%CI 0.5-3.1) and the overall infecting serogroup (Table 3). However, subgroup analyses suggest that Sejroe group infections (OR 4.8, 95% CI 0.9-27.0) and Icterohaemorrhagiae group infections (OR 2.0, 95% CI 0.9-4.3) were more likely to result in CPC compared to serogroup Grippotyphosa. Remarkably, when taking ICU attendance as a marker for severity of acute illness, Sejroe group infections caused milder disease (none of 8 cases attended the ICU) compared to Icterohaemorrhagiae group infections of whom 19% (17/89) needed ICU care (data not shown). This might indicate a potential effect by distinct serogroups on the occurrence of CPC, apparently unrelated to case severity.

**Table 3:** Uni- and multivariate analysis of risk factors for post-leptospirosis complaints.

**Risk factors**	**Available data within the risk categories**	**CPC group**	**OR 95%CI**	**P value**	**PCAC group**	**OR 95%CI**	**P value**
**Univariate analysis**		**N=236**			**N=225**		
Sex	Male	213	1.3 (0.5-3.1)	0.618°	214	4.1 (0.5-33.0)	0.289°°
	Female	22			10		
	Missing	1			1		
Age	≥38 years	117	2.2 (1.3-3.7)	0.003°	114	2.5 (1.4-4.5)	0.002°
	<38 years	119			111		
	Missing	0			0		
ICU	Yes	31	2.8 (1.3-6.3)	0.009°	11	2.2 (0.6-8.0)	0.203°
	No	200			96		
	Missing	5			118		
Dialysis	Yes	24	3.2 (1.3-8.0)	0.01°	20	1.2 (0.4-3.2)	0.763°
	No	211			92		
	Missing	1			17		
Serogroup	Grippo	39	Reference	0.047°	33	Reference	0.827°
	Ictero	92	2.0 (0.9-4.3)		84	0.6 (0.3-1.5)	
	Sejroe	8	4.8 (0.9-27.0)		67	0.7 (0.3-1.7)	
	Pomona	18	0.6 (0.2-2.1)		14	1.0 (0.3-3.6)	
	Other	35	1.1 (0.4-2.7)		7	0.7 (0.1-4.2)	
	Unknown*	44			20		
***Acute injuries** (1985-1993)*		***N=62***			***N=225***		
ARI	Yes	49	1.0 (0.2-3.7)	0.612°°	111	1.6 (0.8-3.1)	0.171°
	No	10			75		
	Missing	3			39		
ALI	Yes	7	1.5 (0.3-7.3)	0.703°°	15	1.1 (0.3-3.7)	0.580°°
	No	44			78		
	Missing	11			132		
Liver injury	Yes	46	6.4 (1.2-33.0)	0.016°	128	1.2 (0.5-2.8)	0.658°
	No	11			33		
	Missing	5			64		
Haemorrhage	Yes	19	0.7 (0.2-2.3)	0.608°	43	1.3 (0.6-2.7)	0.539°
	No	31			101		
	Missing	12			81		
**Multivariate analysis**		**N=236**			**N=225**		
ICU stay	Yes	31	2.0 (0.8-4.8)	0.112		NA	
	No	200					
	Missing	5					
Age	≥38 years	117	2.0 (1.2-3.5)	0.010		NA	
	<38 years	119					
Dialysis	Yes	24	2.3 (0.9-6.2)	0.098		NA	
	No	211					
	Missing	1					

The table presents data from the period 1985 till 2010, unless otherwise indicated

NA: not applicable

Ictero: icterohaemorrhagiae

Grippo: Grippotyphosa

° Chi-square test

°° Fisher’s exact test

Excluded from the analysis

### Factors associated with Persistent Complaints by Active Case finding (PCAC)

In total 321 questionnaires were distributed of which 256 (79.8%) were returned. Data from 31 questionnaires were excluded because the information provided was incomplete (n=28) or contained conflicting data (n=3). This resulted in 225 eligible participants’ questionnaires. The median age was 38 years (IQR 20-46 years) of which 214 were male (95.5%). This is comparable to those participants excluded, i.e. 33 years (23-46 years) and 88 males (91.7%), respectively.

The number of PCAC was 68 (30.2%), and for 65 (95.6%) cases these persistent complaints were specified. Depression-compatible complaints were most frequently reported, such as extreme fatigue, headache and malaise, or complaints that seemed to present persistence, such as myalgia and joint pains (Table 1). Several participants reported back pain and vertigo (3 (4.6%) patients, each), depression and concentration problems (2 (3.1%) patients, each). One participant reported sleep disorders and one reported severe mental alterations. 27 of 65 participants (39.7%) reported ‘other symptoms’, this included typical thoracic pain, perspiration, painful shoulders, stomach pain, and tinnitus.

**Figure 1:**
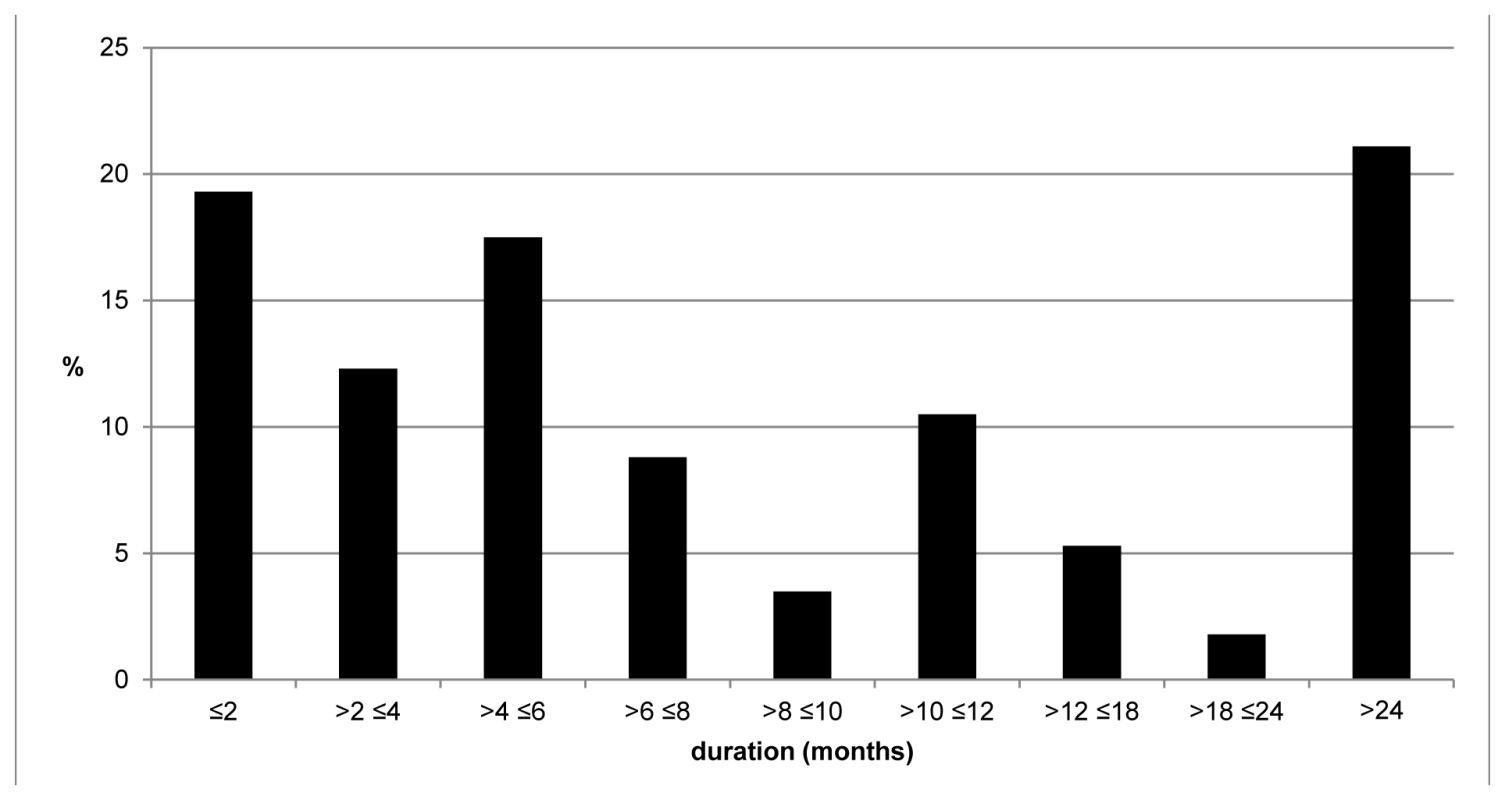
Persistent complaints by active case finding (PCAC) stratified according to months of duration. The bars represent the percentage of the total of reported post-leptospirosis complaints separated according to duration, expressed in periods of months as indicated on the X-axis.

The duration of PCAC was reported for 57 cases, 11 (19.3%) cases reported ≤2 months, and 1 case reported 18-24 months (Figure 1). However, 12 (21.1%) cases had chronic complaints for more than 24 months. Difficulties in returning to work after the acute phase of leptospirosis were reported in 9 out of 68 (13.2%) questionnaires, comprising the inability to work in a dusty environment (1/9), disability to work for 2 months (2/9), bed rest for 2 months (1/9), the need of assistance at the farm for 6 months (1/9), increasing inability to do farmers work (1/9), resigning the job because of disability of the associated labour (1/9) and permanent disability to work (2/9).

Data in Table 3 show that higher age (≥38 years) was positively associated with the occurrence of PCAC (OR 2.5, 95% CI 1.4-4.5), and males trended towards having more complaints than females (OR 4.1, 95% CI 0.5-33.0), although not significant. There was no association between age groups and duration of PCAC or the type of symptoms, although higher age showed a trend towards a higher proportion of myalgia (OR 3.3, 95% CI 0.7-16.4) and physical condition problems (OR 5.3, 95% CI 0.6-44.9).

### ICU stay and persistent complaints

Univariate analysis revealed an increasing trend of post-leptospirosis complaints associated with ICU attendance that was significant for CPC (OR 2.8, 95%CI 1.3-6.3), Table 3.

Furthermore, it seemed ICU attendance and renal injuries were not significantly associated with PCAC. Age was the only risk factor significantly associated with PCAC, therefore only univariate analyses were performed. This suggests that ICU stay as well as the age of ≥ 38, dialysis and liver injury were independently associated with an increase in the odds of complaints (Table 3) in the CPC group but not in the PCAC group.

No significant association was found between ARI, ALI and haemorrhage and persistent complaints in both CPC and PCAC groups (Table 3). Dialysis and age were retained in the multivariate model to control for confounding (P<0.1). The association between ICU stay and age in the univariate model was 2.8 (95% CI 1.3-6.3); while in the multivariate model it was 2.0 (95% CI 0.8-4.8), Table 3. The odds ratio for dialysis decreased from 3.2 (95% CI 1.3-8.0) in the univariate analysis to 2.0 (95% CI 0.8-4.8) in multivariate analysis, suggesting some confounding due to ICU stay and age.

### Agreement between PCAC and CPC groups

The occurrence of persistent symptoms was assessed through two methods, one using active case finding using questionnaires and one using passive finding using routine data from existing archive files. From 50 patients information was available both on CPC and PCAC. When comparing data from overlapping cases in both routes, results of CPC and PCAC corroborated for 32 cases (64%), which implies a slight agreement (Cohen’s kappa = 0.298). Fourteen of the 27 cases with CPC (51.9%) reported prolonged complaints for 2 months or more. On the other hand, four PCAC cases had no report on CPC (S1).

## Discussion

The main objective of this study was to estimate the burden of human leptospirosis in the Netherlands with regard to the frequency and nature of post leptospirosis symptoms as well as the duration of both the acute and persistent phase. In addition, the effects of age, gender, severity of acute illness, including ICU attendance and infecting serogroups were investigated as potential risk factors on the occurrence of post-leptospirosis complaints.

Dutch hospitalized patients had a median stay in the hospital of 10 days and spend 5 days of illness before hospitalization. The total duration of the acute phase thereby consisted of a median 16 days. This concurs with previous data on the duration of acute leptospirosis in the Netherlands and in other parts of the world [3,15]. A limitation of this data is that it concentrates on acute illness from severely ill patients attending the hospital and therefore excludes less severe cases, for which no hospital data were available. However, hospitalization occurred in almost 80% of cases. Moreover, less severe leptospirosis was recognized as such, suggesting a relative high grade of severity for these cases (obviously, patients did seek medical care and the clinician considered leptospirosis), comparable or exceeding that of an influenza-like disease. Taking into account that recovery from a severe influenza-like disease usually requires one to two weeks, ambulant cases are unlikely to considerably reduce the mean duration of acute leptospirosis. Additionally, it is estimated that at least 30% of severe patients in the Netherlands, are missed due to unawareness or mis-diagnosis [3,16]. Denial of effective treatment because of misdiagnosis probably results in a higher severity of complications and a longer duration of illness [17] and hence missed severe cases might well balance the decrease in hospital days caused by ambulant cases.

The duration of hospital stay showed a declining trend toward recent years. Although this does not exclude the increase of less severe forms of leptospirosis, this more likely reflects a general trend in the Netherlands aiming at the reduction of time of hospital attendance [18–20]. This can be partially explained by economic considerations in the Netherlands and can partially be attributed to developments in medical science and improved health awareness in the population [17]. Thus the decline in duration of hospital stay does not reflect a reduction of the burden of the acute phase of disease but rather indicates that its definition based on discharge of the hospital might become less appropriate.

Significant differences were found between the two age categories, with older patients having a longer hospital stay during the acute phase compared to younger patients. This is in accordance with various reports that indicate that increasing age is associated with increasing severity of leptospirosis [21]. This is not unique for leptospirosis but is commonly seen for a variety of infectious diseases in the Netherlands (CBS).

Significant differences were also found between males and females. Over 90% of the confirmed cases were male patients and they spent more time in the hospital than their females counterparts (11 versus 7.5 days). Similarly, the majority of ICU cases were males (89/93) with a median ICU stay of 7 days. This together indicates that males seem to acquire leptospirosis more frequently and develop more severe complications than females. This further substantiates earlier reports that suggested that males are more susceptible to severe leptospirosis than females [13,22]. The assessment of the economic impact of the disease burden is beyond the scope of this paper. However, it should be noted that the vast majority (86%) of severe cases are male patients between 20 and 65 years of age, which is the current working age in the Netherlands. Since females comprise less than 10% of those presenting with leptospirosis, it is likely they will have little effect on the national economic burden.

In 236 eligible participants from a total of 552 records information was collected on persistent complaints in the first month following the acute disease. This was most likely a representative sample, since the median age and sex ratio was comparable for those included and excluded cases. Nearly half of these patients (45.8%) reported continuing complaints one month after discharge from the hospital. This corroborates well with a previous prospective study in Brazil, where complaints were reported in 49% of former hospitalized patients at a follow up of about 20 days [7].

The assessment of PCAC comprised a sample of 225 eligible participants from a total group of 321 patients, with a comparable age and sex in the included and excluded groups. PCAC occurred for one third of the patients with a declining incidence towards a prolonged duration. However, one-fifth of the PCAC patients had complaints that continued more than two years. Complaints were generally depression-like symptoms such as extreme fatigue, malaise, and headache. Physical complaints included myalgia and joint pains affecting mobility. In this sample, only a few files included information on problems in executing ones job. However, it is well conceivable that these depression-like conditions also impacted on the ability to continue working and hence should be expected to constitute a burden to DALYs.

The PCAC study group was conducted from 1985 to 1993 and was contained within the CPC study group. This allowed for a comparison to investigate the association between the two data sets used for these two groups. The presence or absence of complaints was consistent for two-third of the patients who had both PCAC and CPC data. About half of the CPC group patients apparently had recovered from the post-leptospirosis complaints since PCAC was not indicated. However, for four PCAC group patients no CPC was documented. This can be explained by assuming that either the patients or the clinician considered CPC as normal at the ambulant consultation and did not include the data in the report. On the other hand, it is well conceivable that sequelae develop after apparent complete cure from the acute illness as has been reported for leptospirosis- related uveitis [23].

Evidence has consistently shown that serovars Icterohaemorrhagiae and Copenhageni of the Icterohaemorrhagiae group cause severe leptospirosis more often than serovar Hardjo-bovis (Sejroe group) and serovar Grippotyphosa (Grippotyphosa group) [12,14,24,25]. Assuming that sequelae notably occur after traumatic and stressful acute complications, one might expect to find persistent complaints largely corroborating with Icterohaemorrhagiae as the infecting serogroup. However, our data did not show any association between any infecting serogroup and PCAC, although we found an association compared to the Grippotyphosa serogroup, albeit not significant, between CPC and infections with the Icterohaemorrhagiae group. Surprisingly, however, this association was more pronounced with Sejroe as the infecting serogroup. These Sejroe infections are predominantly due to a dairy fever outbreak caused by serovar Hardjo-bovis in the period 1985-1995 [14]. Since complications requiring ICU attendance apparently were absent, we argue that Sejroe group infections cause less severe forms of leptospirosis. Thus, while there seems to be a correlation between the causative infection serotype and severity of illness on one hand, this is not the case between disease severity and the occurrence of persistent complaints on the other hand. Consistently, delayed physical and psychological recovery after Hardjo infection, taking several months to more than a year have been reported by others [26]. Research among randomly selected dairy farms in 1986 and 1987 showed that 63% of persons seropositive for leptospirosis suffered from prolonged excessive fatigue, which led to a partial or complete disability to work [27]. Taking these arguments together, we conclude that less severely ill dairy fever patients do have a relatively high risk for persistent complaints. The reason for this remains unclear, although it is tempting to speculate that the host-dependence of the serovar Hardjo [28] is associated with a greater invasive power of the central nervous system and/or induces other pathways in the host defence responses.

ICU stay is considered to be a traumatic cause of long lasting sequelae [8,9,11,29–31]. In concordance with this, ICU patients had about a twice-higher risk for persistent complaints that was significant for CPC. The risk even increased nearly a 12-fold when combined with dialysis as another stressful event [32].

Although data on the duration of acute leptospirosis and hospital and ICU stay have been presented in previous studies, these are scarce [3]. Hence, the data on incidence, duration of disease and the occurrence of persistent complaints assessed in this paper present a valuable contribution to the current initiatives to estimate the global burden of leptospirosis (WHO-LERG) [2].

This study has some caveats. Numbers, notably of ICU stay and of female cases are low and affect the statistical analyses robustness. Additionally, too few data on the length of ICU stay were available for adequately examining the association ICU stay and PCAC. Although the severity of illness was assessed by analysing several reliable indicators, such as ICU stay, dialysis, ALI and ARI, this study did not use a ‘severity of illness scoring system’ such as APACHE II score or SAPS score [31]. Finally, and probably most importantly, data on sequelae are based on persistent complaints after acute leptospirosis. This does not necessarily imply that these can be fully attributed to leptospirosis. Indeed, persistent complaints also have been reported for other infectious diseases and thus are not unique to leptospirosis. Uniqueness of persistent or chronic symptoms can only be made plausible when including a proper control group, which was not part of this study. Moreover, sequelae, generally consisting of depression-like symptoms, need to be corrected for similar complaints in the whole population in order to clarify that a proportion of the sequelae indeed can be exclusively attributed to the acute disease. We have addressed this point by consulting national databases on population-wide trends in health and lifestyle [33].

Compared to the persistent leptospirosis (CPC and PCAP of 45.8% and 30.2% respectively) the baseline data were much lower, i.e. 18.5% of the general Dutch population did not have a good health with several minor complaints summarized as malaise and likely only for part constitute complaints with a severity that has been reported for persistent leptospirosis. Additionally, there is little correlation between malaise complaints and age in the general population [33], while for leptospirosis such complaints increase with increasing age. Therefore, it is concluded that acute leptospirosis induces persistent and late sequelae that contribute to the disease burden. Reports on chronic leptospirosis usually concern anecdotic case reports. This is one of the few, if not first, systematic reports on the burden of both acute and chronic leptospirosis and as such forms a highly valuable contribution to the assessment of its global burden.

## Supporting Information

Table S1 **Agreement between CPC and PCAC study groups; period 1985 to 1993.**
(TIF)
