# Supplementary figures and images for: Towards the Burden of Human Leptospirosis: Duration of Acute Illness and Occurrence of Post-Leptospirosis Symptoms of Patients in The Netherlands
Source: PLoS One. 2013 Oct 3;8(10):e76549. doi: 10.1371/journal.pone.0076549 (PMC3789694; doi:10.1371/journal.pone.0076549)

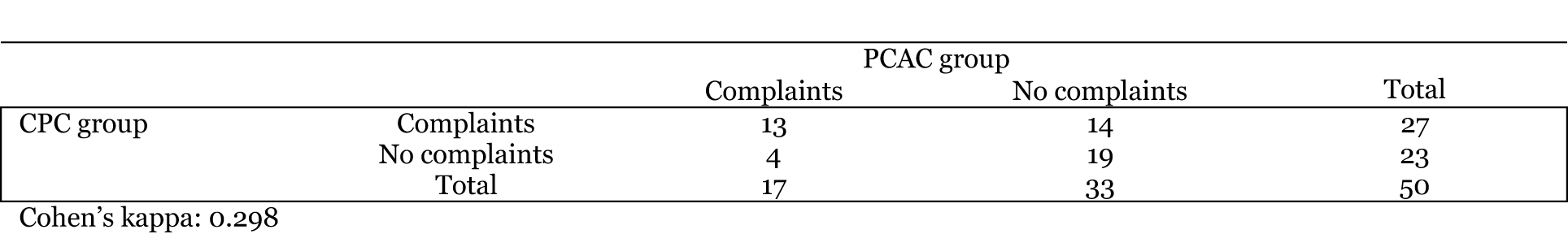

Supplement: Table S1 — Agreement between CPC and PCAC study groups; period 1985 to 1993. (TIF) [file pone.0076549.s001.tif]
